# Addressing Barriers to Health Care Access and Their Impact on Social Cohesion Among Rohingya Refugees and Host Community in Cox’s Bazar, Bangladesh: Protocol for a Qualitative Study

**DOI:** 10.2196/89917

**Published:** 2026-08-14

**Authors:** Samaha Masroor Saqib, Andreas Önver Cetrez, Soorej Jose Puthoopparambil

**Affiliations:** 1 Department of Women’s and Children’s Health, Faculty of Medicine and Pharmacy, Uppsala University, Dag Hammarskjölds väg, Uppsala, 752 37, Sweden; 2 Department of Psychology of Religion, Faculty of Theology, Uppsala University, Uppsala, Sweden

**Keywords:** rohingya refugees, host community, health care provider, health care access, Bangladesh, health inequalities

## Abstract

**Background:**

Access to health care remains a critical issue in protracted forced displacement contexts globally. In Cox’s Bazar, Bangladesh, the co-existence of Rohingya refugees and the host community for many years has generated complex health care dynamics. While humanitarian interventions have increased service availability for refugees, barriers still persist, and disparities in health care access between refugees and host populations exist.

**Objective:**

This study aims to understand the experiences and coping strategies of refugees, host community members, and health care providers involved in accessing and delivering health care services, and to explore how these dynamics influence social cohesion between the two communities.

**Methods:**

The study will use a qualitative design involving focus group discussions (FGDs) with Rohingya refugees and host community members, and in-depth interviews (IDIs) with health care providers in Cox’s Bazar, Bangladesh. Separate FGDs will be conducted with men and women from both refugee and host communities. Participants will be recruited through purposive sampling, complemented with snowball sampling, where required. Each IDI and FGD will be guided by guides developed from prior studies and contextual insights. The anticipated sample includes approximately 3 to 5 FGDs with Rohingya refugees, 3 to 5 FGDs with host community members, and 10 to 12 IDIs with health care providers. Data will be analyzed using Braun and Clarke’s reflexive thematic analysis, and the analysis will be primarily inductive, while also considering the wider social, institutional, and structural conditions that shape participants’ experiences. Since the data will be transferred to Sweden for secure storage, management, and analysis, ethical approval has been obtained from both Sweden and Bangladesh.

**Results:**

Preparatory work began in 2025 following ethical approval, focusing on stakeholder engagement, field coordination, tool refinement, and recruitment planning. Findings are expected to be submitted for publication by 2027.

**Conclusions:**

Guided by a critical realist paradigm, this study is grounded in the understanding that individuals’ lived experiences of accessing and delivering health care are shaped by deeper social, structural, and institutional mechanisms that may not be immediately visible. By integrating the perspectives of refugees, host community members, and health care providers, the study will generate context-specific evidence to inform more equitable and socially responsive health care delivery in Cox’s Bazar and similar humanitarian settings. Additionally, by exploring how disparities in health care access affect perceptions of inequalities, the study will shed light on the broader implications for social cohesion between these coexisting communities. Findings will help inform future strategies to improve equitable health care delivery and policies in displacement-affected regions.

## Introduction

### Background

Globally, the intersection of forced displacement and health care has become a critical arena for examining equity in contemporary public health [1]. As ongoing crises such as conflicts, persecution, and political instability continue to displace millions, host countries face the dual challenge of meeting humanitarian obligations while ensuring adequate health care for both displaced and host populations. These challenges are particularly pronounced in low- and middle-income countries (LMICs), where health systems are already constrained by limited resources, workforce shortages, and competing national health priorities. It becomes more relevant when a large share of displaced individuals are hosted in LMICs [2]. In such contexts, the influx of displaced populations can intensify existing systemic strains, and these intersecting demands can extend beyond logistical and resource-related constraints, giving rise to complex social dynamics that influence perceptions of fairness, belonging, and trust within shared service environments [3,4].

The Rohingya refugee crisis is one of the most pressing humanitarian challenges globally. Following targeted violence and persecution in Myanmar’s Rakhine State, in 2017, more than 740,000 Rohingya refugees had crossed the border into Bangladesh, an LMIC, joining previous waves of displaced populations since 1978 and bringing the total refugee population in Cox’s Bazar to over one million [5-7]. The majority of these refugees reside in various densely populated camps in the Cox’s Bazar district, where the Government of Bangladesh, in coordination with national and international agencies, has mounted a large-scale humanitarian response [8,9]. While these efforts have tried to address the basic humanitarian needs such as food, shelter, water, and health services [6], the scale and protracted nature of the displacement have strained local infrastructure and services, including the public health care system.

Health actors, including government institutions, nongovernmental organizations (NGOs), and international organizations, operate in a highly coordinated yet fragmented environment, with services being provided through both fixed facilities and mobile clinics. Despite these efforts, significant barriers to health care persist, including long waiting times, supply shortages, and financial constraints [10-12]. For the host community, these challenges are compounded by the rapid demographic change and competition for limited resources, with many feeling neglected in the resource allocation process despite facing comparable vulnerabilities to those of the refugee population. These perceptions of inequity have the potential to undermine trust, not only in the organizations responsible for delivering aid but also between the refugee and host populations themselves, thereby posing a risk to social cohesion [13,14].

Previous quantitative studies have documented disease prevalence, service coverage, and health indicators in this context [15-17]. However, there remains a critical need to explore how individuals and providers experience the health care system differently. The few qualitative studies that exist have highlighted some aspects of health or health care access and use, but focus narrowly on one group [18], usually refugees [19,20], without exploring the perspectives of the host community or health service providers. This siloed approach limits our understanding of the broader health system dynamics and the tensions that can arise in shared service environments, especially in protracted displacement contexts. It is also unclear how these tensions may influence social cohesion between refugees and the host community in resource-limited settings [4,21]. This becomes more important as there are increasing calls for integrating refugees into the host community [22,23]. Moreover, there is limited documentation of how frontline workers adapt service delivery strategies, build trust, and negotiate ethical dilemmas while working across both groups, especially when the humanitarian health sector has invested in training and deploying local health care workers [24]. Health care providers' own attitudes and biases can influence patient experiences [25,26].

While our previous quantitative research in Cox’s Bazar has documented measurable differences in health care access between Rohingya refugees and the host community [27], important questions remain about how these differences are understood and experienced in their lives. In our earlier study, we found that Rohingya refugees were more likely than host community members to face financial barriers when accessing health care, but also had higher odds of accessing selected services, including antenatal care, nutrition programs, COVID-19–related information, and formal health care. These findings highlighted that access can be shaped by a complex combination of humanitarian service provision, resource constraints, targeted programming, financial hardship, and differences in how refugees and the host community navigate the health system [27]. However, quantitative findings alone cannot explain how these disparities are perceived by the communities themselves, how they influence experiences of fairness or neglect, or how they shape relations between Rohingya refugees and host community members in shared service environments.

Therefore, this qualitative study extends our previous quantitative work by moving beyond the documentation of health care access disparities to examine the mechanisms, meanings, and consequences underlying those disparities. This qualitative study aims to explore and understand the experiences and adaptive strategies of Rohingya refugees, host community members, and health care providers in accessing and delivering health care services in Cox’s Bazar. This approach is particularly important for informing context-sensitive, inclusive, and equitable health policies and humanitarian strategies. In doing so, the research aligns with the broader goal of global health equity [28], particularly in protracted displacement settings where the boundaries between humanitarian and development responses are increasingly blurred [29,30]. These findings can help humanitarian organizations and government agencies design strategies that improve health care provision for both communities, thereby addressing health needs while also strengthening social cohesion.

### Paradigm

This study will be underpinned by a critical realist paradigm [31], which posits that a reality exists independently of our perceptions, but our understanding of it is always partial, socially influenced, and theory-laden. Critical realism recognizes that a social phenomenon such as experiences of health care access and delivery are shaped by underlying structures, for example, socioeconomic inequalities, institutional practices, and policy frameworks that may not be immediately visible [32]. Within this orientation, the study will examine participants’ accounts of health care access and service delivery not only as descriptions of individual experience, but also as reflections of the social, institutional, and structural mechanisms through which access, perceived fairness, trust, and intergroup relations are shaped in a protracted displacement setting [33,34].

## Methods

### Study Design

We will adopt a qualitative research approach to address the aims of this study. Data collection will include semistructured in-depth interviews (IDIs) with health care providers and focus group discussions (FGDs) with members from the refugee and host community. This design is appropriate given the study’s aim to capture complex, contextual, and subjective experiences. The design and reporting of this study protocol are and will be guided by the COREQ (Consolidated Criteria for Reporting Qualitative Research) [35].

### Study Setting

The study will be conducted in Cox’s Bazar, a coastal district in the Chittagong Division of Bangladesh. Administratively, Cox’s Bazar comprises 8 Upazilas (subdistricts) [36], among which Ukhiya and Teknaf are selected as the study areas. Currently, there are 34 refugee camps which are located primarily in these two subdistricts [37]. The study will be conducted in Rohingya refugee camps in both the Upazilas, and in adjacent host community areas within Cox’s Bazar District. In this study, the host community is referred to as the community living in the catchment area of the health facilities of the refugee camp, that is, the geographical zone from which these facilities draw their patients, ensuring that the study focuses on communities most directly affected by the shared health care system.

### Participants and Sampling

Three participant groups will be targeted: (1) Rohingya refugees aged 18 and above, (2) host community members aged 18 and above, and (3) health care providers (doctors, nurses, and community health workers) serving both populations. All participants must be able to communicate verbally and must demonstrate emotional and cognitive capacity to participate in IDIs or FGDs. Individuals will be excluded if they present with or are diagnosed with mental, neurological, or physical conditions that would prevent meaningful participation, or if they do not consent to having the conversation audio-recorded.

Purposive sampling [38] will be used to achieve maximum variation in age, occupation, and experience. Snowball sampling may also be used, if required, but it will not be the primary recruitment method and will be balanced with other entry points to avoid recruiting only from closely connected social networks. The FGDs will be stratified by gender, with separate male and female FGDs for both Rohingya refugees and host community participants. We anticipate conducting 3 to 5 FGDs each among refugee and host community groups, with 6 to 8 participants per discussion. Approximately 10 to 12 IDIs will be conducted with health care providers. However, the final sample size will be determined based on the concept of “information power,” where data richness and relevance, rather than numerical thresholds, will dictate data saturation [39]. Thus, recruitment will continue while new IDIs or FGDs add important variation, new perspectives, or insufficiently explored experiences related to health care access or social cohesion until the research team judges that the data are sufficiently rich and varied to address the study objectives across the 3 participant groups, and when additional data collection is unlikely to substantially deepen or change the developing analysis.

Eligibility criteria for participation (shown in Table 1) include being aged 18 or older, having accessed (or assisted someone to access) or delivered health care within the past 6 months, and having the cognitive and emotional capacity to provide informed consent and meaningfully participate in discussions. Those with known severe mental health disorders or conditions impairing verbal communication will be excluded to ensure ethical participation and data integrity [40]. Given the sensitive nature of the topic and the socio-political context, sampling will be responsive and adaptive. The team will continually assess and adjust the sample size to ensure depth and richness in thematic development.

Site selection will be guided by the field feasibility, safety considerations, and the need to capture variation in health care access experiences across the participant groups. Within Ukhiya and Teknaf, camps will be selected in consultation with a collaborating research institution in Bangladesh and relevant field-level authorities, taking into account variation in camp location and accessibility. Host community sites will be selected from adjacent unions and villages located near the selected camps and within the catchment areas of health facilities used by both Rohingya refugees and host community members.

**Table 1.** Purposive sampling for the study: participant eligibility.

Participant group and characteristics	Inclusion criteria	Rationale
Refugees		
Status	Registered Rohingya refugee living in the studied camps	To understand the lived experience of displaced individuals receiving camp-based health care
Age	Adults aged 18 years and above	Legal adult age for consent and richer experiential recall
Sex	Male and female participants	To capture gender-based differences in health care experience
Language or communication	Able to converse in Rohingya dialect or Bangla	For effective FGDs^a^ participation with interpreter assistance
Mental capacity	No known or diagnosed severe mental health condition; able to provide informed consent	Ensures participants can comprehend and meaningfully engage in the FGD process, maintains ethical standards, and data quality
Health service use	Has experience of using or assisting a close friend or relative in using any health care service in the past 6 months	Ensures relevance and recency of the experiences being shared
Host community		
Status	Resident of communities bordering refugee camps in Cox’s Bazar	To explore perceived impacts and access challenges from the host perspective
Age	Adults aged 18 years and above	Legal adult age for consent [41] and richer experiential recall
Sex	Male and female participants	Gendered differences may shape health care access and behavior
Language	Able to communicate in Bangla	For effective FGDs participation and analysis
Mental capacity	No known or diagnosed severe mental health condition; able to provide informed consent	Ensures participants can comprehend and meaningfully engage in the FGD process, maintains ethical standards, and data quality
Health service use	Has experience of using or assisting a close friend or relative in using any health care service in the past 6 months	Ensures participants have recent experiences to share
Health care providers		
Role	Health care providers serving refugee and host populations (eg, doctors and nurses)	To understand differences in frontline service provision challenges along with coping strategies
Location	Employed in health facilities within or near Cox’s Bazar refugee camps (eg, NGO^b^ clinics and hospitals)	Ensures experience is grounded in the context of both the humanitarian and host setting
Experience	Minimum 6 months working with both groups	Ensures sufficient familiarity with community health dynamics
Language	Able to communicate in Bangla or English	Facilitates meaningful IDIs^c^ without language barrier

a FGD: focus group discussion.

b NGO: nongovernmental organization.

c IDI: in-depth interview.

### Recruitment Strategy

Recruitment will be facilitated through partnerships with a health research institution in Bangladesh that has longstanding involvement in the region. This research institution has been actively involved in implementing and evaluating health interventions targeting both Rohingya refugees and the host community, such as infectious disease surveillance, maternal and child health, nutrition, and health systems strengthening. However, to reduce their influence and selection bias, they will not make final decisions about who participates in the study. Their task will be limited to assisting in stakeholder engagement (connecting with community health workers and NGO field staff) and field logistics, by leveraging their extensive field networks, established rapport with key stakeholders, and operational familiarity within both the refugee camps and host community settlements.

Community health workers, NGO field staff, and camp focal points will assist in identifying potential participants who meet the eligibility criteria. For the Rohingya refugee and host community members, trusted community members and local leaders (eg, teachers, religious leaders) will be approached to support community entry and to facilitate introductions where appropriate. For health care providers, recruitment will include different professional cadres and will be carried out through direct contact with different facility types, such as hospitals, primary health care centers, and NGO clinics operating within and around the camps. Where necessary, initial participants may also be asked to refer others using snowball sampling to reach individuals with relevant experiences who may not be easily identified through formal channels.

Participants will be approached in person or via phone if needed, depending on accessibility and safety considerations. The study team will ensure the use of culturally appropriate communication strategies, such as explaining the study’s purpose and voluntary nature by providing translated information letters and consent forms in Bangla and the Rohingya dialect (where appropriate), and using community interpreters to ensure understanding. For example, interpreters familiar with local norms will help explain confidentiality and informed consent in a manner that is both linguistically and culturally appropriate. These trained interpreters will assist in explaining study details and obtaining informed consent. Privacy and confidentiality will be emphasized throughout the process to ensure participants feel safe and respected.

To promote inclusion and ethical engagement, particular attention will be given to recruiting women and underrepresented voices, including older adults and persons with disabilities. Recruitment will continue until information power is deemed sufficient across all participant groups.

### Data Collection

Data collection will involve both IDIs and FGDs, conducted face-to-face in the field. These methods were selected to capture individual narratives of health care providers and collective experiences regarding health care access and delivery among Rohingya refugees and the host community. The separation of FGDs by population group is intended to promote open dialogue and minimize power imbalances or social discomfort.

To ensure consistency across sessions, all IDIs and FGDs will follow semistructured IDI (Multimedia Appendix 1) and FGD guides (Multimedia Appendix 2) that have been developed based on gaps identified in previous studies in Cox’s Bazar and other similar forced displacement contexts, covering core topics related to health care access, service delivery, adaptive strategies, perceptions of fairness, trust, intergroup relations, and social cohesion. The guides will include open-ended questions, designed to elicit detailed responses and to allow comparable topics to be explored across participant groups, while allowing flexibility for participants to introduce topics of importance to them. Follow-up probes will be used to encourage elaboration, clarification, and reflection.

IDIs are expected to last approximately 45‐60 minutes, while FGDs are expected to last approximately 60‐90 minutes, depending on participant availability, group dynamics, and the depth of discussion. Each FGD will include approximately 6‐8 participants to allow sufficient diversity of views while maintaining a manageable group size for discussion. For each FGD, at least two trained team members will be present: one facilitator and one note-taker. Where interpretation is required, a trained interpreter fluent in the Rohingya dialect will support communication. Female facilitators or field team members will be used for women’s FGDs where feasible, to improve comfort and culturally appropriate participation. All facilitators, note-takers, and interpreters will receive training before data collection on the study objectives, IDI and FGD guides, informed consent procedures, confidentiality, management of group dynamics, neutral probing, avoidance of leading questions, distress response, and secure data handling. Training will also include practice sessions using the IDI and FGD guides to support consistency across sessions.

All IDIs and FGDs will be done by the first author, except for the FGDs with Rohingya refugees, where the FGDs in Rohingya dialect will be conducted by trained field researchers fluent in the Rohingya dialect, and supported by the first author. Each IDI or FGD will be audio-recorded with the participant’s consent to ensure accuracy in transcription and analysis. Field notes will be maintained throughout the data collection process to capture nonverbal cues, contextual information, setting characteristics, group dynamics, and the researcher’s reflections. These notes will enrich the interpretation of the data and provide critical insights during the analysis phase. Throughout the data collection phase, the research team will engage in continuous reflection and methodological adaptation in response to field realities, such as participant dynamics, contextual sensitivities, or logistical constraints. Weekly team check-ins and debriefs will be conducted to review experiences in the field, discuss emerging challenges, and make necessary adjustments to the guides or procedures to enhance data quality.

Given the multilingual nature of the study, particular attention will be given to transcription, translation, and preservation of meaning. IDIs conducted in Bangla or English will be transcribed verbatim in the language of data collection. FGDs conducted in the Rohingya dialect will first be translated and transcribed into Bangla by trained field researchers who are familiar with both the Rohingya dialect and Bangla, as well as the local Cox’s Bazar context. Where English translations are required for analysis and reporting, Bangla transcripts will be translated into English by SMS (first author), who is from Bangladesh and is fluent in Bangla and English.

Selected sections of translated transcripts will be checked against the original audio recordings and Bangla transcripts, particularly sections containing culturally specific terms, unclear expressions, emotionally sensitive accounts, or quotations likely to be used in reporting. Rohingya or Bangla words or expressions that do not have an exact equivalent in English will be translated in a way that preserves the participant’s intended meaning, with explanatory notes added in brackets where needed. Translation-related decisions and interpretive choices will be documented in analytic memos and considered during coding and theme development.

### Data Analysis

#### Overview

Data will be analyzed using Braun and Clarke’s [42] reflexive thematic analysis, a widely recognized and flexible approach suitable for exploring qualitative data. This method acknowledges the researchers’ active role in identifying patterns of meaning and constructing themes through a reflexive and iterative process. It allows both semantic and latent meanings to be explored across the accounts of Rohingya refugees, host community members, and health care providers, while remaining sensitive to the social and contextual conditions shaping health care access and service delivery.

The analysis will proceed in six recursive phases [42], which include (1) data familiarization, (2) generating initial codes, (3) searching for themes, (4) reviewing themes, (5) defining and naming themes, and (6) writing the report. Analysis will be iterative and inductive, allowing themes to emerge organically from the data. A software will support systematic coding and data management, such as organizing transcripts, coding data extracts, storing analytic memos, retrieving coded segments, comparing patterns across participant groups, and supporting the development of thematic maps will be used.

#### Data Familiarization

The analysis will begin with repeated reading of the interview and FGD transcripts alongside the audio recordings and field notes. This will help to become closely familiar with participants’ accounts, including how Rohingya refugees, host community members, and health care providers describe health care access, service delivery, and interactions within the health system. During this stage, reflective notes will be written to capture early impressions, recurring issues, and points of contrast between participant groups. Particular attention will be given to initial observations related to barriers to care, perceptions of fairness, trust in health care providers and institutions, and experiences that may influence relationships between the refugee and host communities.

#### Generating Initial Codes

The coding process will begin with in vivo coding, in which participants’ own words and expressions will be used as initial codes to preserve the meanings embedded in their accounts. This approach will help capture how refugees, host community members, and health care providers describe health care access, fairness, trust, and intergroup relations in their own terms. These initial codes will then be reviewed, refined, and developed into analytical codes that capture broader patterns across the data; that is, meaningful segments of the transcripts will be coded inductively, allowing codes to emerge from the data rather than from a predefined coding framework. Codes will capture both explicit descriptions and underlying meanings in participants’ narratives. Coding will be reviewed and refined as new transcripts are analyzed, and similar codes will be grouped where appropriate.

#### Searching for Themes

Once the initial codes are generated, the third phase will focus on identifying broader themes, patterns of meaning that are significant to the research objectives. This involves examining how different codes may combine to form overarching narratives, tensions, or shared experiences. At this point, a preliminary thematic map will be developed to visualize how themes relate to each other.

#### Reviewing Themes

During this phase, preliminary themes will be reviewed and refined in relation to the full dataset. Themes may be merged, divided, or discarded to ensure internal coherence and clear distinction between them. This process also verifies that the thematic map accurately reflects the data and addresses the research question.

#### Defining and Naming

Themes: once the themes have been reviewed, each theme will be clearly defined and named to capture its central meaning and contribution to the analysis. This stage will involve identifying what each theme reveals about participants’ experiences of health care access and service delivery, and how these experiences relate to perceptions of fairness, trust, inclusion, or tension between groups. The analysis will move beyond description by interpreting the significance of each theme within the broader displacement and health system context of Cox’s Bazar.

#### Producing the Report

In the final phase, the themes will be developed into a coherent analytic account that addresses the study aims and research questions. Consistent with reflexive thematic analysis, writing will be approached as an integral part of analysis, through which the meaning, boundaries, and interrelationships of themes are further refined. Each theme will be presented through a structured narrative, supported by selected pseudonymized quotations that illustrate key patterns within the data. The final report will move beyond descriptive summary to interpret how participants’ accounts illuminate experiences of health care access, perceptions of fairness and trust, and the ways in which health care delivery may shape relationships between refugees, host community, and health care providers.

Social cohesion will be examined through several interrelated dimensions; for example, trust in health care providers, humanitarian actors, and institutions; perceived fairness in the distribution and accessibility of health care services; intergroup relations between refugees and host community; expressions of solidarity, mutual support, or shared vulnerability; and narratives of resentment, neglect, competition, or conflict linked to health care access and service delivery [22,43,44]. Social cohesion will be used as sensitizing concepts rather than as fixed deductive coding categories to guide attention to theoretically relevant issues while still allowing meanings, patterns, and themes to be inductively identified through participants’ narratives about their experiences of accessing, receiving, or delivering health care [45]. During coding and theme development, particular attention will be paid to how participants describe fairness or unfairness in service provision, trust or mistrust toward health care actors, perceptions of preferential treatment, everyday interactions between refugees and the host community in health facilities, and accounts of cooperation, resentment, blame, or tension. These dimensions will not be imposed as fixed categories, but will sensitize the analysis to how health care access is socially interpreted by different groups. This approach will allow the study to examine how health care disparities are not only experienced as service-related barriers, but also as social experiences that may influence perceptions of belonging, exclusion, legitimacy, and coexistence between Rohingya refugees and the host community. Since reflexive thematic analysis will be used for this study, the analysis will first remain inductive and grounded in participants’ narratives, and then move to a later interpretive stage in which the identified themes are considered in relation to possible underlying mechanisms. Thus, the analysis will not only describe perceived barriers, coping strategies, and service delivery experiences, but will also explore how and why these experiences may shape trust, perceived fairness, intergroup relations, and social cohesion between refugees and the host community [46].

The first 3 phases (data familiarization, generating initial codes, and searching for themes) will be led by the PhD student SMS (first author), who will be directly involved in reading transcripts, coding data, and identifying initial patterns of meaning. The subsequent phases (reviewing, defining, and naming themes, and producing the report) will be conducted together by the SMS and the main supervisor SJP (last author). To strengthen analytic depth and transparency, a subset of transcripts from each participant group will also be read by the main supervisor SJP and discussed with the research team. The purpose of involving more than one researcher is not to calculate inter-coder reliability, but to support reflexive engagement with the data, question emerging interpretations, and consider alternative explanations [47]. The subsequent phases, including reviewing, defining and naming themes, and producing the report, will be conducted collaboratively by SMS and SJP, with methodological and contextual input from AÖC and colleagues from the collaborating research institute where appropriate. The process will be reflexive, with the researcher actively reflecting on their positionality, assumptions, and influence on interpretations. Team discussions and memo-writing will support analytic rigor and transparency. Throughout all phases, the analysis will be iterative, meaning earlier phases may be revisited as new insights emerge.

### Field Notes

Field notes will be used as a critical complementary data source during both data collection and analysis. These notes will be maintained by researchers and trained field staff and will serve to document contextual details surrounding the IDIs and FGDs, including physical setting, nonverbal cues, group dynamics, interruptions, and the emotional tone of participants. Field notes will also be used to record the researcher’s observations, impressions, and methodological reflections throughout the fieldwork process [48].

By paying close attention to both the environment and the interactional aspects of data collection, the field notes will enhance the richness and depth of the dataset and support a nuanced interpretation of the findings. Additionally, the notes will promote self-awareness and help researchers reflect critically on their positionality, preconceptions, and potential sources of bias, thereby strengthening the study’s overall rigor and transparency [48]. Regular briefings will be held between the first and last authors during data collection, as a reflexive exercise and to discuss various aspects of data collection.

### Ethical Considerations

Ethical approval for conducting this study has been received from the Swedish Ethical Review Authority (Dnr 2025-05315-01) and the Ethical Review Committee of the International Center for Diarrhoeal Disease Research, Bangladesh (PR-24153).

Prior to each session of FGD or IDI, informed consent will be obtained, and participants will be informed of the risks associated with the study and reminded of their right to withdraw at any point without any consequences. For participants with limited or no literacy, written informed consent will be obtained through a thumbprint procedure. The participant information sheet and consent form will be read aloud in Bangla or the Rohingya dialect, as appropriate, by a trained researcher or the interpreter. The researcher will check comprehension by asking whether the participant understands the purpose of the study, the voluntary nature of participation, the right to refuse or withdraw, the confidentiality procedures, the use of audio-recording, and the fact that participation will not affect access to health care, humanitarian assistance, or any other services.

IDIs and FGDs will be conducted in private, neutral locations that are convenient and safe for participants, such as community centers, NGO offices, or quiet areas within camp or village settings. To ensure privacy, locations that are quiet and less crowded will be selected, and it will be ensured that only authorized participants are present during data collection. For FGDs, participants will be asked to respect the privacy of others and not repeat outside the group what is shared during the discussion. The facilitator will also remind participants that they do not need to share personal or sensitive information that they feel uncomfortable disclosing in front of others. This is particularly important because focus groups can create risks of over-disclosure, social discomfort, or unintended disclosure of sensitive experiences [49]. If a participant becomes distressed at any point in time during the data collection, the facilitator will pause the discussion, check whether the participant wishes to continue, skip the question, take a break, or stop participation entirely. Arrangements will be made with local NGOs and health care providers to provide support for any participant who might experience emotional distress during their participation.

To appreciate participants’ time and contribution, a modest token of appreciation (eg, snacks and drinks) will be provided, consistent with ethical research practices and local customs. No financial compensation will be made. The research team will ensure that the incentive does not create undue influence or pressure to participate by clearly explaining during the consent process that participation is entirely voluntary.

All collected data will be transferred to Sweden and will be securely stored on Uppsala University’s password-protected servers as well as encrypted storage devices, and physical notes, including signed consent forms and field notes, will be kept in locked storage. Access will be restricted to authorized research team members only, in compliance with data protection and ethical standards. Confidentiality will be maintained by pseudo-anonymizing transcripts and removing identifying details. Audio recordings will be securely stored as per the relevant national regulations. All the materials will be retained for 10 years in accordance with Uppsala University data management requirements and then securely deleted.

### Reflexivity

The research team recognizes that their backgrounds, values, and prior experiences may influence data interpretation. Reflexivity will therefore be practiced throughout the research process to minimize bias and enhance analytical depth. This includes ongoing critical self-reflection, documentation of decision-making processes, and discussions among team members regarding how their perspectives may shape data collection, analysis, and interpretation. Reflexive practice is particularly relevant in a study situated in a complex humanitarian context, where power dynamics and positionality require careful consideration [42,50].

The first author, SMS, is a Bangladeshi female PhD student in migration and health at Uppsala University with prior training and experience in medicine and global health. Her Bangladeshi background and familiarity with the broader health system and sociocultural context may facilitate rapport with host community members, health care providers, and local collaborators. At the same time, her position as a university-based researcher affiliated with a European institution, and as an outsider to the Rohingya refugee community, may influence how participants perceive her role, what they choose to disclose, and how power relations are negotiated during data collection. These positionalities will be considered during recruitment, facilitation, interpretation, and reporting. The co-authors bring complementary disciplinary and methodological perspectives. AÖC’s background in psychology of religion, migration, acculturation, refugee psychosocial health, culture, religion, and meaning-making may sensitize the analysis to psychosocial and relational dimensions of displacement, but may also shape how issues such as trust, identity, belonging, and social relations are interpreted. SJP’s background in migration and global health, health systems, health inequalities, forced displacement, and previous research on health care access among Rohingya refugees and the host community in Cox’s Bazar may provide contextual familiarity, while also requiring attention to how prior knowledge of the setting and earlier findings may influence expectations during analysis. The collaborating Bangladeshi research institution and local field researchers will provide contextual and linguistic insight, particularly in relation to local health systems, community dynamics, and the Rohingya dialect. However, the research team also acknowledges that local field staff, interpreters, and institutional partners may hold their own social positions and relationships within the study setting, which may shape participant recruitment, communication, and interpretation.

The positionalities will be discussed during team meetings and documented through reflexive memos to examine how disciplinary assumptions, institutional roles, prior research experience, and local relationships may shape recruitment, data collection, coding, and theme development. Reflexive memo-writing will be integrated throughout data collection and analysis [50]. During data collection, memos will document impressions from IDIs and FGDs, group dynamics, ethical concerns, translation-related issues, and possible effects of researcher identity, institutional affiliation, or local partnerships on participant interaction. During analysis, memos will be used to record coding decisions, emerging interpretations, assumptions, uncertainties, and alternative explanations. Reflexive memo-writing will contribute to the audit trail and will be revisited to examine how the team’s backgrounds and prior experiences may shape interpretation, and to ensure that themes remain grounded in participants’ accounts [50,51].

### Trustworthiness and Limitations

#### Overview

We will strive to ensure the trustworthiness of the study through strengthening credibility, transferability, dependability, and confirmability [51,52].

#### Credibility

Credibility will be enhanced through multiple strategies [53]. Triangulation will be applied across data sources and participant groups: the perspectives of Rohingya refugees, host community members, and health care providers will be compared to identify common themes or discrepancies, strengthening the consistency of our findings [53,54]. We will further ensure the credibility of the study by actively engaging and building trust within the community through interaction and presence in the field (eg, spending time in camps and local clinics before and during data collection). This sustained engagement will allow the research team to become familiar with local norms, languages, and routines, helping to reduce social distance and power imbalances between researchers and participants [51].

#### Dependability

To ensure dependability, a detailed audit trail will be maintained, documenting all stages of the study, from data collection tools and sampling decisions to transcription, coding, and theme development [52,53]. A code-recode strategy will be applied, along with refining the codebook for consistency. Regular team discussions throughout data collection, analysis, and writing will further ensure that interpretations are consistent and not shaped by a single researcher’s perspective [51,55].

#### Confirmability

Confirmability will be enhanced through a detailed audit trail documenting analytic decisions, ensuring that interpretations can be traced directly to raw data [51,53]. We will also incorporate verbatim quotations from participants in the results, allowing readers to see the basis of the results in participants’ own words. Reflexive practices, where researchers document and reflect on their own assumptions and positionality, will help minimize subjective influence throughout data collection and analysis [53-55].

#### Transferability

Transferability relates to the extent to which the study’s findings can be applied or be useful in other contexts [53]. Our study is situated in a specific context, the Rohingya refugee camps and adjacent host community in Cox’s Bazar, Bangladesh. To facilitate transferability, we will provide “thick description” of the research setting, participants, and processes [53,56]. This means our reporting will thoroughly describe the context of the camps (eg, living conditions, health care system structure), the characteristics of participants (eg, sociodemographic details), and the circumstances under which data were collected. By delivering detailed contextual information, we will aim to enable readers to assess the similarity of our context to other environments [53,57].

Although qualitative research does not aim for reliability in the statistical sense, consistency in the analytical process is essential to ensure trustworthiness. Reliability in this study will be addressed by maintaining a clear audit trail that documents all analytical steps and researcher reflections throughout the coding and theme development phases, ensuring transparency in how codes and themes are derived. Analytical procedures will include regular peer discussions and reflexive memo writing to record analytic decisions. This approach ensures that the analysis process remains transparent and logical for other independent researchers to be able to trace and reach comparable interpretations of the data [58].

## Results

As this is a study protocol, no empirical findings have been reported in this manuscript. Ethical approval was obtained from both Sweden and Bangladesh in 2025. Following ethical approval, the preparatory phase, including stakeholder engagement, refinement of data collection tools, field coordination, and recruitment planning, began in 2025 as well. The findings are expected to be disseminated through peer-reviewed journal publications by 2027 as well as through conference presentations, and potential policy-oriented outputs for relevant stakeholders involved in health care delivery and humanitarian programming in Cox’s Bazar.

## Discussion

### Anticipated Findings

This study aims to shed light on the lived experiences of Rohingya refugees, host community members, and health care providers in accessing and delivering health care in the humanitarian setting of Cox’s Bazar, Bangladesh. In a context where the refugee situation has become protracted, the study will also explore how health care access disparities influence social cohesion among the groups. To the best of our knowledge, no previous studies have simultaneously examined the perspectives of refugees, host community, and health care providers within a single analytical framework. By integrating these interrelated viewpoints, this study offers a more comprehensive understanding of health care access and delivery in displacement settings, thereby contributing new insights to the field of migration and health.

Previous studies in Cox’s Bazar have documented health needs, service delivery challenges, and disparities affecting Rohingya refugees and the surrounding host community [27,59,60]. However, much of the existing evidence is either quantitative or focused on one participant group, limiting understanding of how health care access is socially interpreted across the refugee-host interface [61,62]. By including refugees, host community, and health care providers, this study will allow comparison across multiple perspectives within the same health care environment and may help explain how shared or unequal access to services affects perceptions of fairness and intergroup relations.

A key strength of this study is the inclusion of 3 participant groups: Rohingya refugees, host community members, and health care providers. This design will allow triangulation across service users and providers and will support a more comprehensive understanding of health care access and delivery. The use of separate FGDs for men and women will help create a more comfortable environment for discussing sensitive issues, while purposive and maximum variation sampling will support the inclusion of diverse experiences across age, location, caregiving role, service-use experience, professional cadre, and facility type. The use of reflexive thematic analysis will allow the study to remain grounded in participants’ narratives while also interpreting how wider social, institutional, and structural conditions shape health care access and social cohesion.

In case of limitations, as with qualitative research generally, the findings will not be statistically generalizable to all Rohingya refugees, host community members, or health care providers in Cox’s Bazar. However, the study will provide rich contextual understanding that may be transferable to similar protracted displacement settings, supported by a detailed description of the study context, participants, and analytic process. Additionally, recruitment through local networks, field staff, and community contacts may introduce selection bias by making some participants more visible than others. To mitigate this, the study will use multiple recruitment channels, limit gatekeeper involvement in final participant selection, monitor participant variation during recruitment, and document recruitment decisions. Another limitation may be specific to FGDs, where the data may be affected by group dynamics, social desirability, and limits to confidentiality. These risks will be addressed through gender-stratified FGDs, clear explanation of confidentiality limits, careful facilitation, and encouragement not to disclose sensitive information that participants do not feel comfortable sharing in a group setting. Moreover, translation of the transcripts may affect meaning. To reduce this risk, trained bilingual personnel will conduct transcription and translation, selected transcript sections will be checked against recordings, and translation decisions will be documented in analytic memos.

The findings can generate important insights for health care practitioners, humanitarian actors, and policymakers working in crisis-affected settings. By understanding the diverse challenges and adaptive strategies of different groups, the study will help identify both gaps and strengths in current service delivery. Ultimately, this research can inform more equitable, culturally sensitive, and context-specific interventions aimed at improving health care access and trust among displaced and host populations in similar low-resource displacement settings.

### Dissemination of Findings

The findings from this study will be disseminated through peer-reviewed articles published in international academic journals in the fields of global health, migration, and health systems research. Additionally, results will be shared at relevant national and international conferences, and presented in policy briefs to inform stakeholders involved in health care provision in humanitarian settings. Options to conduct stakeholder engagement workshops, based on the results, will also be explored.

## Supplementary material

10.2196/89917 Multimedia Appendix 1 Interview guide.

10.2196/89917 Multimedia Appendix 2 Focus group discussion guide.

## Abbreviations

COREQ: Consolidated Criteria for Reporting Qualitative Research
FGD: focus group discussion
IDI: in-depth interview
LMIC: low- and middle-income country
NGO: nongovernmental organization
