# Supplementary material for: Addressing Barriers to Health Care Access and Their Impact on Social Cohesion Among Rohingya Refugees and Host Community in Cox’s Bazar, Bangladesh: Protocol for a Qualitative Study
Source: JMIR Res Protoc. 2026 Aug 14;15:e89917. doi: 10.2196/89917 (PMC13475931; doi:10.2196/89917)
Supplement: Supplementary file 1 [file resprot-v15-e89917-s001.docx]

**Appendix 1**

**Draft Interview Guide (Healthcare providers): Understanding Healthcare Experiences**

**Note:**

The current interview guide is a preliminary version, designed to provide an initial framework for our qualitative research. It will be refined and updated based on key factors identified in the quantitative part of the project, where the study aims to identify differences in healthcare access between Rohingyas and host community along with identifying factors associated with it. This follows an emergent design approach where key results from the quantitative study, in addition to other key aspects, would be further explored through qualitative methods so that a more comprehensive understanding of the situation could be developed.

**Interview Guide**

***Before & after the Interview notes sheet***

Number of interview:

Place: Date: Language: Duration of interview:

Interviewer/Researcher: Transcription: from hand notes / from audio recording

Position/group of participant interviewed:

**Observations and notes about the interview**

*Please take notes on the circumstances of the interview, for a comprehensive understanding of the context. Think about: How did you get the appointment? Where does the interview take place? In a public / private / office space? Are there other people around? Is everyone on time or late, is anyone pressed for time? How do researcher and interviewee position themselves towards each other, what is the atmosphere?*

**Observations about the location of the interview**

*Please take any notes about what you observe at the location of the interview that offers insights: the people, things and technologies that you observe, and any interactions between people. If it is useful, visual aids (drawings, photos with permission).*

**Introduction of the Interview**

**Instructions:**

- Repeat the information in the Information Letter
- Ensure that the environment is comfortable, and the participant feels safe sharing sensitive information.
- Remain neutral and refrain from influencing the participant’s opinions.
- Emphasize that the information shared will remain confidential

**General Topic & Interview Setup:**

**Welcome and Introduction**

- Introduce the interviewer and note-taker.
- Explain the purpose of the interview:
  "We are here to learn about your experiences regarding healthcare provision to Rohingyas and host and understand how you manage to overcome challenges while providing healthcare."

The main themes we would like to discuss:

- General situation of healthcare services.
- The interviewee's role and connections.
- Difference in providing healthcare in case to host vs Rohingyas
- Opportunities and barriers to providing healthcare
- Overcoming these barriers
- Suggestions for improving healthcare services
  - Mention the duration of the interview.
  - Reassure the participant that his/her views are important, and there are no right or wrong answers.

**Informed consent:**

**Informed Consent**:

I have provided the Consent Form to you earlier, and I have printed copies for you to review [hand over the documents].

Here are the key points to keep in mind:

- **Voluntary Participation**: Your participation in this study is entirely voluntary. You can withdraw at any point in time, without providing any reason and consequences.
- **Data Protection**: All the information you share will be pseudonymized to protect your identity and securely stored at the Uppsala University's secure server, which can be accessed only through personal authentication. Only researchers within the project will have access to the pseudonymized data.
- **Publication**: When we publish or present our findings, we will ensure that no personal information that could be used to identify you will be made available.
- Additionally, I would like to ask for your permission to record our conversation so that we can accurately transcribe it later. The recordings will **not** be shared with any unauthorized personnel and will be archived for 10 years after the project’s completion while maintaining strict protection and security measures throughout the storage period.

Do you have any questions about this? [give a couple of minutes for the respondent to reflect upon the informed consent and ask questions, if any]. If everything is clear, could you please fill in the Consent Form?

If you agree, I’ll start the recording now.

**Interview Questions:**

**0. Warm-up**

- Can you please tell me a little about your professional background?
- What is your role at this clinic/healthcare centre and how long have you been working here?
- From your experience, can you please give me an overview of the health services provided at this clinic?

**1. Defining/Situating Healthcare Services**

- **Understanding healthcare services:**
- How would you define healthcare services for Rohingyas and the host community here in [place]?
- When and how did these services start?
- What services are available to both of the groups?
- **Healthcare process:**
- Describe the typical pathway for a Rohingyas or host community member from identifying a disease symptom at home to the first point of contact to accessing healthcare.
- What people, organizations, and technologies are involved?
- **Probing questions:**
- Who/What are the key actors behind healthcare services?
- Who are the target populations for the services?
- What are the involvements of Rohingyas and the host community?
- How well are the services provided here accessed by Rohingyas and the host community?

**2. Specific questions:**

- **Experiences Providing Healthcare:**
  - Can you describe your role and responsibilities in providing healthcare services in [place]?
  - What have been your experiences in providing healthcare to Rohingyas and the host community? Are there any differences?
- **Healthcare Needs**:
  - What types of healthcare services (e.g., emergency, maternity, mental health) do you think are most difficult to access?
  - What makes these services difficult to access?
  - Are there any services that you feel are lacking or insufficient?
- **Challenges in Healthcare Provision**:
  - How successful are Rohingyas and host communities in seeking care when they need and to receive right care?
    - Any differences between the groups?
  - What do you think are the main barriers for Rohingyas and host to seek care at your clinic?
  - What are the main challenges you face in providing healthcare services to Rohingyas and the host population?
  - Have you faced any issues related to resource allocation, infrastructure, or staffing?
- **Mitigation Strategies:**
  - What measures are taken to address these challenges?
    - By you or your organization
    - By the communities
    - By other actors
  - Are there any best practices or successful strategies you would like to share?
  - How do you coordinate with other organizations/agencies/government to improve healthcare delivery?
- **Suggestions for Improvement:**
  - What recommendations do you have for improving the healthcare system in [place]?

**3. Summary and closure**

We are getting towards the end of our conversation. Thank you so much for your perspective on the healthcare services.

- **Additional Comments:**
  - Do you have any additional comments you would like to make or issues not addressed that you would like to mention?
- Ensure to thank the interviewee for their time and insights.
- Arrange for follow-up if necessary.
- Ask for suggestions for other potential participants.
- Inform the participant where to get information on the outcomes of the project.
