# Supplementary material for: Addressing Barriers to Health Care Access and Their Impact on Social Cohesion Among Rohingya Refugees and Host Community in Cox’s Bazar, Bangladesh: Protocol for a Qualitative Study
Source: JMIR Res Protoc. 2026 Aug 14;15:e89917. doi: 10.2196/89917 (PMC13475931; doi:10.2196/89917)
Supplement: Supplementary file 2 [file resprot-v15-e89917-s002.docx]

**Appendix 2**

**Draft** **Focus Groups Discussion Guide (Rohingyas and host communities):**

**Understanding Healthcare Experiences**

**Note:**

The current Focus Groups Discussion (FGD) guide is a preliminary version, designed to provide an initial framework for our qualitative research. It will be refined and updated based on key factors identified in the quantitative part of the project, where the study aims to identify differences in healthcare access between Rohingyas and host community along with identifying factors associated with it. This follows an emergent design approach where key results from the quantitative study, in addition to other key aspects, would be further explored through qualitative methods so that a more comprehensive understanding of the situation could be developed.

**FGDs will be held separately for Rohingyas and host communities and for women and men.**

**FGD Guide**

***Before & after the FGD notes sheet***

Number of FGD:

Place: Date: Language: Duration of FGD:

Facilitator: Transcription: from hand notes / from audio recording

Position/group of participants:

**Observations and notes about the FGD:**

*Please take notes on the circumstances of the FGD, for a comprehensive understanding of the context. Think about: How did you get the appointment? Where does the discussion take place? In a public / private / office space? Are there other people around other than the participants? Is everyone on time or late, is anyone pressed for time? How do researcher and participants position themselves towards each other, what is the atmosphere?*

**Observations about the location of the FGD:**

*Please take any notes about what you observe at the location of the FGD that offers insights: the people, things and technologies that you observe, and any interactions between people. If it is useful, visual aids (drawings, photos with permission).*

**Introduction of the FGD**

**Instructions:**

- Repeat the information in the Information Letter
- Encourage respect and open communication.
- Ensure everyone gets a chance to speak.
- Ensure that the environment is comfortable, and participants feel safe sharing sensitive information.
- Be mindful of cultural sensitivities.
- Remain neutral and refrain from influencing the participants’ opinions.
- Emphasize that the discussion will remain confidential

**General Topic & FGD Setup**:

**Welcome and Introduction**

- Introduce the facilitator(s) and note-taker(s).
- Explain the purpose of the discussion:
  "We are here to learn about your experiences regarding healthcare access and understand how you manage healthcare challenges."

The main themes we would like to discuss:

- General situation of healthcare services
- The participant's role and connections
- Opportunities and barriers to seeking healthcare
- Differences in seeking healthcare for host communities vs. Rohingyas
- Overcoming these barriers
- Suggestions for improving healthcare services
  - Mention the duration of the FGD.
  - Reassure participants that their views are important, and there are no right or wrong answers.

**Informed Consent**:

I have provided the Consent Form to you earlier, and I have printed copies for you to review [hand over the documents].

Here are the key points to keep in mind:

- **Voluntary Participation**: Your participation in this study is entirely voluntary. You can withdraw at any point in time, without providing any reason and consequences.
- **Data Protection:** All the information you share will be pseudonymized to protect your identity and securely stored at the Uppsala University's secure server, which can be accessed only through personal authentication. Only researchers within the project will have access to the pseudonymized data.
- **Publication:** When we publish or present our findings, we will ensure that no personal information that could be used to identify you will be made available.
- Additionally, I would like to ask for your permission to record our conversation so that we can accurately transcribe it later. The recordings will **not** be shared with any unauthorized personnel and will be archived for 10 years after the project’s completion while maintaining strict protection and security measures throughout the storage period.

Do you have any questions about this? [give a couple of minutes for the respondent to reflect upon the informed consent and ask questions, if any]. If everything is clear, could you please fill in the Consent Form?

If all the participants agree, I’ll start the recording now.

**Discussion Questions:**

**0. Warm-up**:

- Briefly introduce yourself and ask participants to do the same.

Can you briefly introduce yourself (your name, background, and how long you've been part of this community)?

- Ask participants to share their general experiences with healthcare services in [place].

**1. Defining/Situating Healthcare Services**:

- **Understanding Healthcare Services**:
  - How would you describe healthcare services available for Rohingyas and the host community here in [place]?
  - How do you usually access healthcare services in this area?
  - What services are available to you?
- **Healthcare Process**:
  - Describe the typical process involved in seeking healthcare for [Rohingyas/host community member] from the first point of contact to receiving care.
  - What people, organizations, and technologies are involved?

**2. Specific Questions for Each Group**:

***For Rohingyas****:*

- **Experiences Seeking Healthcare**:
  - Can you describe the circumstances that led to your displacement within your country? How has your displacement affected your access to healthcare compared to before you were displaced?
  - What has been your experience in accessing healthcare services in [place]?
  - How do you find information about available healthcare services?
  - Have some of you discovered other resources that help find information?
  - How easy or difficult is it to access healthcare services when you need them?
- **Healthcare Needs**:
  - As a group, what type of healthcare services do you feel are essential for you right now?
  - What types of healthcare services (e.g., emergency, maternity, mental health) are most difficult to access?
  - Is this a shared experience among the group, or do some of you find different services more challenging to access? What makes these services difficult to access?
  - Are there any services that any of you feel are lacking or insufficient?
  - Since 2017, large numbers of Rohingyas have started arriving. Do you think there is any difference in the availability or quality of healthcare before 2017 in comparison to the current time?
- **Barriers and Challenges**:
  - If you reflect upon when you were successful to receive the healthcare you needed, why was it successful?
  - Has the experience been similar for the rest of you or different?
  - What are some of the current opportunities available for Rohingyas, and members of the host community to receive the care they need?
  - Do others think that these opportunities are accessible to everyone?
  - What are the main barriers you encounter in getting the healthcare you need?
- Have you faced any difficulties related to language, transportation, cost, or other such factors when accessing healthcare?
- Are there cultural or gender-related barriers in accessing healthcare services?
  - Have others in the group faced similar barriers, or have your experiences been different?

- **Mitigation Strategies**:
  - What steps have you taken to overcome these challenges?
  - For those of you who have encountered similar issues, have you found similar/different ways to cope or overcome these challenges?
  - Have any of you encountered any healthcare initiatives specifically targeted at Rohingyas? If so, how effective have they been?
  - Are there any community or organizational support systems that have been helpful to any of you?
  - How effective do you find the local government initiatives aimed at supporting Rohingyas’ healthcare needs?
  - Do others agree to it?
- **Suggestions for Improvement**:
  - What do you think could be done to improve access to healthcare for both the host community and Rohingyas?
  - Do others have any additional suggestions?

***For Host Community****:*

- **Experiences seeking Healthcare**:
  - What has been your experience in accessing healthcare services in [place]?
  - How do you find information about available healthcare services?
  - Have some of you discovered other resources that help find information?
  - How easy or difficult is it to access healthcare services when you need them?
- **Healthcare Needs**:
  - As a group, what type of healthcare services do you feel are essential for you right now?
  - What types of healthcare services (e.g., emergency, maternity, mental health) are most difficult to access?
  - Is this a shared experience among the group, or do some of you find different services more challenging to access? What makes these services difficult to access?
  - Are there any services that you feel are lacking or insufficient?
- **Barriers and Challenges**:
  - If you reflect upon when you were successful to receive the healthcare you needed, why was it successful?
  - Has the experience been similar for the rest of you or different?
  - What are some of the opportunities available for Rohingyas/host communities to get the care needed?
  - What are the main barriers you encounter in getting the healthcare you need?
    - Have you faced any difficulties related to transportation, cost, or other such factors when accessing healthcare?
    - Are there gender-related barriers in accessing healthcare services?
  - Have others in the group faced similar barriers, or have your experiences been different?
- **Mitigation Strategies**:
  - What steps have you taken to overcome these challenges?
  - For those of you who have encountered similar issues, have you found similar/different ways to cope or overcome these challenges?
  - Have any of you encountered any healthcare initiatives specifically targeted at the local community? If so, how effective have they been?
  - Are there any community or organizational support systems that have been helpful to any of you?
  - How effective do you find the local government initiatives aimed at supporting the local community’s healthcare needs?
  - Do others agree to it?
  - Have you noticed any changes in the quality or availability of healthcare services since the arrival of the Rohingyas in [place]?
- **Suggestions for Improvement**:
  - What do you think could be done to improve access to healthcare for both the host community and Rohingyas?
  - Do others have any additional suggestions?

**3. Summary and Closure**:

We are getting towards the end of our discussion. Thank you so much for your perspective on the healthcare services.

- **Additional Comments**:
  - Do you have any additional comments you would like to make or issues not addressed that you would like to mention?
- Ensure to thank the participants for their time and insights.
- Consider whether additional FGDs or interviews are needed based on the discussion outcomes.
- If needed, plan follow-up meetings with participants or other relevant stakeholders
- Ask for suggestions for other potential participants.
- Inform them where to get information on the outcomes of the project.
